# Gut Microbiota, Probiotics, and Their Interactions in Prevention and Treatment of Atopic Dermatitis: A Review

**DOI:** 10.3389/fimmu.2021.720393

**Published:** 2021-07-14

**Authors:** Zhifeng Fang, Lingzhi Li, Hao Zhang, Jianxin Zhao, Wenwei Lu, Wei Chen

**Affiliations:** ^1^ State Key Laboratory of Food Science and Technology, Jiangnan University, Wuxi, China; ^2^ School of Food Science and Technology, Jiangnan University, Wuxi, China; ^3^ National Engineering Research Center for Functional Food, Jiangnan University, Wuxi, China; ^4^ (Yangzhou) Institute of Food Biotechnology, Jiangnan University, Yangzhou, China; ^5^ Wuxi Translational Medicine Research Center and Jiangsu Translational Medicine Research, Institute Wuxi Branch, Wuxi, China

**Keywords:** gut microbiota, probiotics, atopic dermatitis, immune response, effective substances

## Abstract

Atopic dermatitis (AD) is a public health concern and is increasing in prevalence in urban areas. Recent advances in sequencing technology have demonstrated that the development of AD not only associate with the skin microbiome but gut microbiota. Gut microbiota plays an important role in allergic diseases including AD. The hypothesis of the “gut-skin” axis has been proposed and the cross-talk mechanism between them has been gradually demonstrated in the research. Probiotics contribute to the improvement of the intestinal environment, the balance of immune responses, regulation of metabolic activity. Most studies suggest that probiotic supplements may be an alternative for the prevention and treatment of AD. This study aimed to discuss the effects of probiotics on the clinical manifestation of AD based on gut microbial alterations. Here we reviewed the gut microbial alteration in patients with AD, the association between gut microbiota, epidermal barrier, and toll-like receptors, and the interaction of probiotics and gut microbiota. The potential mechanisms of probiotics on alleviating AD *via* upregulation of epidermal barrier and regulation of immune signaling had been discussed, and their possible effective substances on AD had been explored. This provides the supports for targeting gut microbiota to attenuate AD.

## Introduction

Atopic dermatitis (AD) is an inflammatory skin disease characterized by recurrence, dry skin, erythema and itchiness. The incidence of AD gradually increases with the development of industrialization and urbanization, affecting 15-30% of children and 10% of adults all over the world (1, 2). The clinical symptoms will disappear with growing up in some children with AD, but about 1/2 of children may develop into allergic asthma and 2/3 of children have a risk of allergic rhinitis in the future. The process is called the “atopic march” (3). The scratching induced by intense itchiness leads to skin barrier destruction, which disturbs the immune responses and microbial ecology in the local area and makes patients fall into the circle of “itch-scratch-severe itch”. The itch and recurrence of AD result in a poor quality of sleep, which may be closely associated with the inferiority complex, anxiety, depression, and other psychological diseases in patients with AD based on the brain-skin connection (4). Additionally, it brings a great economic burden to patients due to the long-term treatment for AD and significantly decreases the life quality of their families (5).

The causes of AD are complex and include genetic and environmental factors. Genetic linkage analysis has been identified AD locus on chromosomes 1q21, 17q25 and 20p (6). Furthermore, evidence has been shown that loss and mutations in the gene encoding filaggrin are closely related to AD onset and development (2). Although genetics play an important role in the onset of AD, changes in environmental factors are significantly associated with the increase in prevalence in recent years. Individuals with AD are commonly stimulated by allergens including pollen, dust mite and animal dander around them (7, 8). Skin flora, especially, *Staphylococcus aureus* and *Malassezia* blooms in the lesions results in more severe AD clinical symptoms (9, 10). The pathogenesis mechanism of atopic dermatitis has not been fully demonstrated but T helper type 2 (Th2) - and Th17-skewed immune dysregulation is predominant in the acute phase and chronic phase, respectively (11). Air pollutions, such as polycyclic aromatic hydrocarbons, have been reported to cause Th2 cell-related skin disorders including atopic dermatitis (1). Interleukin (IL) 4 and IL-13 are excessively released after Th2 cell activation and increase immunoglobulin (Ig) E class switching and specific IgE production in B cells (12). The specific IgE binds with the high-affinity receptor FcϵRI expressed by mast cells and basophils, resulting in degranulation of these cells and release of inflammatory mediators and inducing clinical symptoms of AD (13). IL-31, as the product of Th2 cells and immature dendritic cells, activates IL-31 receptor A/oncostatin M receptor to stimulate itch and neuronal outgrowth (14). It has been identified as a critical cytokine involving neuroimmune communication and thus affects the development of pruritus closely associated with nerves in AD patients. The humanized monoclonal antibody against IL-31 receptor A, Nemolizumab, has been reported to result in a significant decrease in pruritus in patients after a 16-week intervention (15). These studies suggest that immune response balance is a critical factor to protect the host from suffering AD. Therefore, regulation for immune response is an effective approach to alleviate atopic dermatitis in patients.

Intestinal microecology is a dynamic and unique ecosystem and affected by diet, living habits and mental stress. The imbalance of gut microbial diversity and composition causes intestinal microecological disorder and results in the shifts of gut microbial metabolism and immune responses. These alterations are closely associated with physiological and pathological activities and important for human health. The maintenance for structural diversity of gut microbiota resists the invasion of pathogen bacteria and reduces the nutritional competition between the potentially harmful bacteria and commensal bacteria. Gut microbiota involved in short-chain fatty acid (SCFA), amino acid, vitamin and bile acid metabolism induces the mature of the innate and adaptive immune system (16). Therefore, the gut microbiota is a potential target for regulating immune responses in the host. With the development of sequencing technology, the correlation between gut microbiota and human diseases including allergic asthma, atopic dermatitis, is revealed in many studies (17–19). The “gut-skin” axis has been proposed and is recognized as a new target to prevent and treat AD. Gut and skin have several similar characteristics and are parts of the overall immune and endocrine systems (20). They are the major compositions of mucosal immunity and directly contact the environmental antigens including foods, commensal bacteria and pathogens. The development of gut diseases is commonly accompanied by cutaneous lesional manifestations and this implies the association between them may affect each other’s states (21). Therefore, targeting gut microbial alterations may be an alternative to regulate immune responses and ameliorate cutaneous health in AD patients. Probiotics and/or prebiotics, as the common regulator for gut microbiota, have been used to alleviate AD clinical symptoms, but with controversial outcomes (positive or ineffective). This is related to the complex interaction between immune response, gut microbiota, and metabolic activity in the host. With a focus on gut microbial alteration, this review discusses the beneficial role of probiotics in the prevention and treatment of AD and the possible underlying mechanisms.

## The Association Between Gut Microbial Diversity, Composition, and AD

Numerous studies have shown that the development of allergic diseases such as asthma and AD is closely associated with changes in gut microbial diversity and composition (22–25). Over 1000 different species reside in the gastrointestinal tract (26), and the number of bacterial cells is about 10 times larger than that of eukaryotic cells in the human body (27). The bacteria have evolved with the human, and it becomes a mutualistic relationship. Therefore, gut microbiota may involve in the development of certain diseases, and the role of gut microbiota is worth exploring in the development of AD. Before birth, microbial compositions have been found in the placenta and meconium, suggesting microbial colonization in early life (28). Following birth, gut microbiota and mucous membranes begin to establish and have been affected by delivery modes such as natural birth or caesarean section (29). *Lactobacillus*, *Prevotella* and *Sneathia* spp. are dominant bacterial communities in the gut of naturally delivered infants, resembling their own mother’s vaginal microbiota, but the microbial communities in caesarean delivery infants are similar to skin microbiota, dominated by *Staphylococcus*, *Corynebacterium* and *Propionibacterium* spp (30), implying the shaping role of delivery mode in the diversity and structure of the initial microbiota. After that, the gut microbial diversity increases rapidly and dietary factors including breast- and formula-fed become the important perturbations for shaping gut microbial diversity and composition. *Lactobacillus* and *Bifidobacterium* are dominant gut microbiota in breast-fed infants at 12 months of age, but *Roseburia*, *Clostrium* and *Anaerostipes*, that belonging to *Clostirdia*, are enriched in the gut microbiota of no longer breast-fed children (31). *Lactobacillus*, *Bifidobacterium* and *Bacteroides* can degrade oligosaccharides from breast milk into small sugars and utilize them to obtain an advantage for growth (32). Therefore, they are the most abundant bacterial communities in the gut of breast-fed infants. *Enterococci* and *Clostridia* are dominant bacteria in formula-fed infants (33), and the intestinal tract contains fewer bacterial cells and more species than in breast-fed infants (34). At 3 years of age, gut microbial composition toward a more stable shift and resemble that of the adult (31).

Bacterial diversity and composition are closely associated with the onset and development of various diseases such as acute infective diarrhea, constipation, obesity, and depression (35–38), underlining the importance of bacterial diversity and colonization in early life for future health. **Table 1** shows gut microbial alteration in patients with AD. Compared to healthy individuals, gut microbial diversity decreased, and the relative abundances of the beneficial microbes such as *Lactobacillus*, *Bifidobacterium* significantly reduced but the proportions of *Escherichia coli*, *Clostridium difficile* and *Staphylococcus aureus* increased in patients. Especially, gut microbial colonization and alteration were demonstrated prior to any clinical manifestations in early life, indicating gut microbial dysbiosis as one of the causes of AD (54). Infants with less gut microbial diversity seem to be susceptible to atopic dermatitis. A cross-sectional study among 1440 children showed that the α diversity of gut microbiota was closely associated with a decreased risk of eczema (55). The α diversity was not different in adult patients suffering from allergic asthma comparing the healthy controls (56). Although the relative abundance of bifidobacteria was reduced, *Bifidobacterium adolescentis* species prevailed within the bifidobacterial population (56). The results showed bifidobacterial composition, especially the proportion of *B. adolescentis*, had special effects on the development of allergic disease. A clinical trial showed that the diversity of *Bifidobacterium* species in allergic infants was similar to non-allergic infants (57). However, in a study from rural Japan infants, allergic infants had a higher abundance of *B. catenulatum* and *B. bifidum* than healthy controls in different stages of age (58). These controversial outcomes further show that the association between allergic disease and gut microbiota is complex and maybe not be restricted to *Bifidobacterium* species. In early life, regulation of gut microbial diversity and composition may reduce the onset and development of allergic symptoms including AD. Therefore, this is an alternative to reduce the adverse reactions of drugs for AD.

**Table 1:** Changes in the gut microbiota of patients with atopic dermatitis.

Type of Study	Nation/Year	Changes in Gut microbiota	Reference
Children, incident AD (n=62)	Estonian, Swedish; 1999	The fewer lactobacilli in the gut of allergic children, higher aerobic bacteria, coliforms, and *Staphylococcus aureus versus* the nonallergic children in the two countries	(39)
Infants at high risk of atopic diseases (n=76)	Finland; 2001	Atopic subjects had more *Clostridia* and fewer bifidobacteria than nonatopic subjects	(40)
Infants, incident AD (n=44)	Estonian, Swedish; 2001	Compared to healthy infants, fewer enterococci and bifidobacteria in the gut of allergic babies. Allergic infants had higher clostridia, *Staphylococcus aureus* and *Bacteroides*.	(41)
Minor patients with AD (n=30), healthy control subjects (n=68, sex-matched)	Japan; 2003	The proportion of *Bifidobacterium* was lower and *Staphylococcus* was higher in patients with AD than that in healthy subjects	(42)
Infants with atopic symptoms (n=957)	Netherlands; 2007	The presence of *Escherichia coli* and *Clostridium difficile* was associated with a higher risk of developing eczema	(43)
Infants with eczema (n=37) and controls (n=24)	United Kingdom, New Zealand; 2008	*Bifidobacterium pseudocatenulatum* was associated with eczema	(44)
Healthy infants (n=20), infants with atopic eczema (n=15)	Swedish; 2008	Alpha diversity indicators were significantly less in infants with atopic eczema than that in healthy infants	(45)
Patients, incident allergic symptoms (n=47)	Swedish; 2009	The relative abundances of *Lactobacillus rhamnosus*, *L. casei*, *L. paracasei*, *Bifidobacterium adolescentis* and *Clostridium difficile* were decreased in allergic children	(46)
Infants with eczema, (n=20), healthy control subjects (n=20)	Switzerland; 2012	Infants with eczema had lower diversity and a lower diversity of *Bacteroidetes*, *Bacteroides* and *Proteobacteria*	(47)
Infants at high risk of allergic disease (n=98)	Australia; 2012	Gut microbial diversity was lower in infants with eczema compared to infants without eczema	(48)
Patients with AD (n=90), healthy control subjects (n=42)	Korea; 2016	The proportion of *Faecalibacterium prausnitzii* was increased in patients with AD	(49)
Healthy infants (n=66), infants with AD (n=63)	Korea; 2018	Bacterial cell amounts were lower and the relative abundances of *Akkermansia muciniphila*, *Ruminococcus gnavus*, and *Lachnospiraceae bacterium* 2_1_58FAA were decreased in infants with AD than in control infants	(50)
Patients with AD (n=23), controls (n=58)	Brazil; 2020	*Clostridium difficile* was associated with AD, and fewer *Lactobacillus* and more bifidobacterial in patients with AD	(51)
Patients with AD (n=44), healthy control subjects (n=49)	China; 2021	Alpha diversity decreased in patients with AD than healthy subjects. *Blautia*, *Parabacteroides*, *Bacteroides ovatus*, *Porphyromonadaceae*, and *Bacteroides uniformis* were increased but *Clostridium* and *Prevotella stercorea* were reduced in patients with AD	(52)
Patients with AD (n=19), other allergic diseases patients (n=20)	China; 2021	The relative abundances of *Bacteroidetes*, *Bacteroidales*, *Bacteroidia*, *Romboutsia*, and *Sutterella* were significantly increased in patients with eczema	(53)

## Regulation of Gut Microbiota on Immune Responses in AD

The “hygiene hypothesis” suggesting that decreased early life microbial exposure and diversity result in loss of immunological tolerance and this is being linked to an increased prevalence of allergic diseases in the urban environment (59, 60). Gut microbiota is the most important component of microbial exposures. Therefore, gut microbial communities affect shaping the host immune development, and dysbiosis of gut microbiota is closely associated with immune disorders (61–63). Throughout the lifespan, the host’s immune system is constantly regulated by gut microbiota. The maternal gut microbial alterations in pregnancy affect the early postnatal immunity of offsprings. Comparing to the offsprings of germ-free mice, gestation colonization with *Escherichia coli* HA107 significantly altered the numbers of postnatal intestinal leukocytes in offsprings and regulated the development of the innate immune system in early life (64). Furthermore, the interactions of gut microbiota with T cells and B cells can lead to systemic outcomes that are distal to the site of the gut. For example, the strains belonging to *Clostridia* induce expansion and differentiation of regulatory T cells (Treg) and alleviate the clinical symptoms of colitis and allergic diarrhea in mice (65). Segmented filamentous bacteria induce the T helper 17 cells (Th17) in the small intestinal lamina propria to drive autoimmune arthritis (66).

A human intervention trial of manipulating urban environmental biodiversity showed that skin and gut microbial diversity of children in nature-oriented daycare centers were increased and this was closely associated with an overall more healthy immune system (67). The ratio of IL-10:IL-17A was increased in plasma samples of these children. The decrease in IL-17A expression was related to and decreased *Romboutsia* and *Dorea*, increased *Anaerostipes*, and higher *Faecalibacterium* Otu00007 in the gut. Gut microbial diversity contributes to the education of the immune system and decreases the prevalence of immune-mediated diseases such as allergies. Additionally, gut microbial colonization promoted the development of microbiota-T cells in the thymus *via* migration of microbial antigens from the gut to the thymus by intestinal dendritic cells (DCs) (68). This not only expanded T cells but increased the capability of thymic T cells to identify gut microbiota and pathogens. It further indicates that gut microbial colonization affects the development of adaptive immune responses and educates the immune system. Therefore, gut microbial alterations are closely associated with the immune responses and play a crucial role in the development of diseases involved in aberrant immune functions.

### Role of Epidermal Barrier in AD

As a systemic disease, AD may have the aberrant barrier function across multiple organ sites including skin, lung and gut. In an epithelial barrier dysfunctions study in AD, epidermal barrier disruption led to allergen sensitization and pathogens colonization. This induced inflammatory response and increased barrier breakdown at distant sites such as the gut and respiratory tract (69). It is suggested that there is a crosstalk mechanism between skin and gut, and thus gut microbial alteration may be associated with epidermal barrier function in AD. In AD, the epidermal barrier may be an important component of the innate immune system because it protects from the invasion of pathogens and allergens and prevents water loss in the skin. Filaggrin (FLG) is an important epidermal protein, and FLG deficiency results in the epidermal barrier defect and increases the risk of the microbiome and virus invasions. It has been demonstrated that FLG deficiency is closely associated with AD and plays a crucial role in the pathogenesis of AD (70). In FLG-deficient (flg^-/-^) mice model, AD symptoms were induced using calcipotriol. Comparing to wild-type mice, the flg^-/-^ mice exerted severer clinical symptoms characterized by increased ear thickness, mast cells and CD3^+^ T cells infiltration, and the level of thymic stromal lymphopoietin, interleukin (IL)-4, IL-6 and IL-13 (71). This is implied that FLG is an important predisposing factor in the pathogenesis of AD. In a genetic correlation study consisting of 386 whole-genome sequencing samples, there was a significant association between FLG function mutation and age of onset of AD (72). *L. plantarum* LM1004 significantly improved the AD-like symptoms, decreased Th2 and Th17 cell transcription factor levels, and increased the transcription factors of Treg and Th1 cells, galactin-9 and FLG (73). This is implied that there is an interaction between probiotics, gut microbiota, and the epidermal barrier. Furthermore, the important feature of AD is an itch, which contributes to damage of the epidermal barrier. Surfaces of skin, lung and gut can act as a shared immunological interface, and environmental stimulation such as gut microbial alteration affects interactions of immune responses among them. This immunological interface as part of the mucosal membrane is the first line to combat infection. Innate lymphoid cells (ILCs) play a key role in the homeostasis and pathology of mucosal membranes and affect the interactions of the “gut-lung” axis. It has been identified that inflammatory type 2 ILCs from the intestine are recruited to the lung by IL-25 and mediate type 2 immune responses (74). Gut inflammation and gut barrier leakage increased the activation of skin epithelial cells and recruitment of T cells to the skin in patients with Omenn syndrome, and this exacerbated the skin inflammation (75). This had been verified in Rag2^R229Q^ mice which simulated the clinical symptoms of Omenn syndrome. These results further identify skin, lung and gut share the immunological interface and it mainly consists of the mucosal membrane from these sites. Therefore, the integrity of the epidermal barrier is essential for maintaining the immune responses in the skin.

### Toll-Like Receptors Signaling in AD

**Figure 1:**
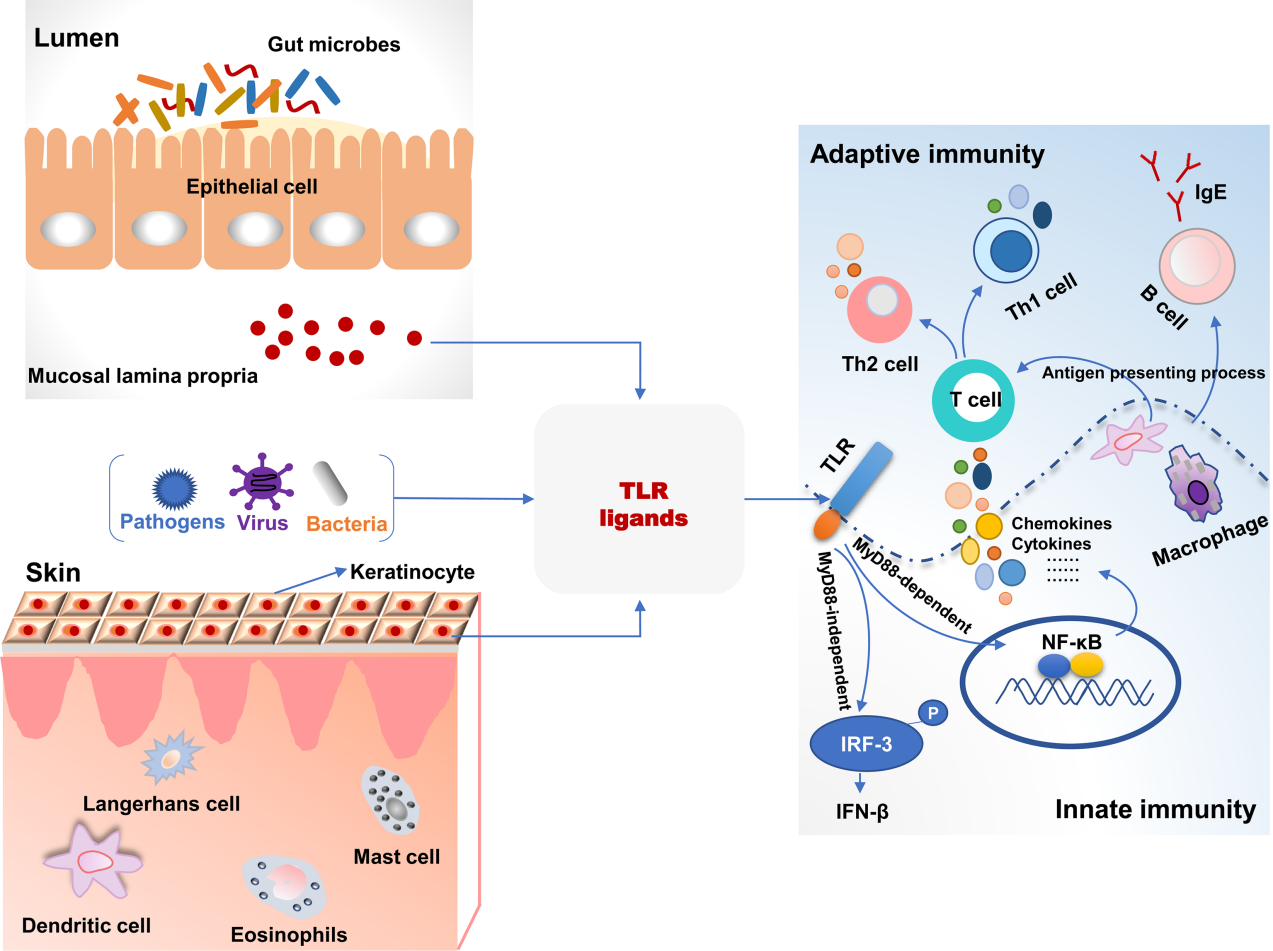
The association of toll-like receptor signaling and immune responses in the intestine and skin. TLR ligands from bacteria, viruses, and pathogens were recognized and activated TLR signaling pathways, which bridged the innate and adaptive immunity in the intestine and skin. TLR, toll-like receptors; MyD88, myeloid differentiation factor 88; P, phosphorylation.

Toll-like receptors (TLRs), a superfamily of pattern-recognition receptors bridge innate and adaptive immunity (**Figure 1**). TLRs are a class of transmembrane non-catalytic proteins and can recognize molecules with conserved structures from microorganisms. These molecules are known as the pathogen-associated molecular pattern (PAMP) such as lipopolysaccharide, peptidoglycan and zymosan. When TLRs bind PAPM, they initiate a signal transduction cascade to activate innate immune responses to eliminate pathogens that break through the skin or mucosa barrier (76). Most TLRs (TLR1, TLR2, TLR4, TLR5, TLR6, TLR10, TLR11 and TLR12) are expressed on the cell surface to recognize PAMPs, but TLR3, TLR7, TLR8 and TLR9 are intracellular to detect nucleic acids (77). As yet, 13 and 10 members of TLRs have been identified in mice and humans, respectively, and their respective ligands also have been revealed (78). The skin harbors various cells expressing TLRs and directly expose to microbes and pathogens in the environment. Therefore, invading pathogens-inducing aberrant TLRs responses may result in skin diseases including AD (79). *S. aureus* colonizes skin lesions of AD patients and can be recognized by TLR2 due to its cell wall components. Compared to healthy volunteers, the expression of TLR2 was decreased on Langerhans cells (LC) in AD patients with high colonization by *in situ* analysis. TLR2 ligand induced maturation and migratory activity of LC and decreased IL-6 and IL-10 production of skin samples from AD patients (80). This suggested that TLR2-mediated immunoregulation signal pathways had been impaired in AD patients. Additionally, macrophages are known to express TLR2 and accumulate in acute and chronic stages of AD in skin lesions. Compared to healthy controls, macrophages from peripheral blood monocytes of AD patients expressed decreased TLR2 and pro-inflammatory cytokines including IL-6, IL-8 and IL-1β after TLR2 ligands intervention (81). In primary human keratinocytes, TLR2 agonists such as *S. aureus*-derived peptidoglycan and Pam3CSK4 significantly improved the tight junction barrier and increased the expression of tight junction proteins. Therefore, the epidermal barrier in AD patients was restored after a TLR2 agonist intervention. TLR2^-/-^ mice also exerted a delayed barrier recovery indicating that TLR2 signaling played a critical role in epidermal barrier integrity (82).

It has been studied that TLR2 rs5743708 and TLR4 rs4986790 polymorphisms are associated with susceptibility to AD (83). In neonates, the incidence of AD was significantly associated with twofold lower TLR4-mediated IL-10 production and resulted in an impaired Th1 type polarizing immune response (84). Furthermore, the single nucleotide polymorphisms (SNPs) related to oxidative stress and inflammation indicating that there was a close association between TLR2, TLR4, and TNF and traffic-related air pollution, and this revealed the gene-environment interactions in the development of AD (85). In TLR4^-/-^ mice, hapten (2,4-dinitrochlorobenzene)-induced AD symptoms and Th2-type inflammatory responses were more severe than wild-type mice and increased the migration of DCs into draining lymph nodes (86). This indicated that TLR4 mediated immune responses associated with AD development. TLR4 ligands increased IL-23 production in skin lesions and resulted in the migration of skin DCs that induced IL-22 expression by naïve CD4^+^ T cells. IL-22 increased keratinocyte proliferation and inflammatory infiltration from Th22 cells in AD mice (87). These studies suggest TLR4 activation contributes to the balance between Th1- and Th2-type immune responses and is one of the targets to treat AD symptoms.

Itch is an important symptom of AD and is associated with TLRs signaling pathways. TLR3 is expressed by small-sized primary sensory neurons and plays a role in modulating sensory neuronal excitability and central sensitization. TLR3 knockdown alleviated pruritus in wild-type mice. In TLR3^-/-^ mice, excitatory synaptic transmission was impaired, and scratching behaviors were significantly decreased after histamine and pruritogens challenges (88). This demonstrated the potential anti-itch role of TLR3 in AD. House dust mite (HDM) is a common allergin and is related to exacerbation of AD. HDM-induced Th2-type immune responses were closely associated with the expressions of IL-25 and IL-33 *via* activation of TLR1 and TLR6 signaling (89). Additionally, in a total of 1063 children cohort study, prenatal contact with farm animals and cats significantly decreased the risk of incidence of AD. This was closely associated with increased expression of TLR5 and TLR9 in cord blood (90). Collectively, TLRs signaling pathways play a critical role in innate immune responses in the development of AD, and impaired TLRs signaling leads to an aberrant balance between Th1- and Th2-type immune responses.

### Association Between TLRs and Gut Microbiota

Gut microbiota and their metabolites can be recognized by TLRs, and the interactions between bacteria and TLRs contribute to systemic immune homeostasis (**Figure 1**) (91). Perturbations in gut microbiota lead to the invasion of microbes and their metabolites into circulation and affect pathological symptoms of the distant site organs such as the brain, liver, kidney, lung, and skin *via* TLRs signaling pathways. In a cohort study with 957 children, there was a significant multiplicative interaction between TLR4 SNP rs10759932 and *E. coli* regarding allergic sensitization in the first 2 years of life (92). It demonstrated the effect of TLR4 genetic variations on allergy development in early life and the modulating role of gut microbe in immune responses in relation to TLRs signaling. An evaluation of gut microbiota and innate immune responses in IgE-associated eczema showed that *Ruminococcaceae* in fecal samples was lower in atopic eczema infants than that in healthy controls and was negatively related to TLR2-induced IL-6 and TNF-α. *Enterobacteriaceae* (a genus of Proteobacteria phylum) was negatively related to TLR4-induced TNF-α, and α-diversity of *Bacteroidetes* and *Actinobacteria* were lower in atopic eczema infants *versus* the controls (93). The administration of a food allergen increased specific IgE and histamine levels and induced allergic symptoms in TLR4-mutant or -deficient mice. However, after antibiotic treatment, gut microbial composition and structure were disturbed in TLR4 wild-type mice, and they were susceptible to the induction of food allergy like the TLR4-mutant mice (94). It indicated that microbes and TLRs signaling were necessary for the development of the immune system. Dysbiosis of gut microbiota results in immune disorder and increases the risk of allergy. TLRs are not only able to recognize invading pathogens and provoke immune responses but play a critical role in the cross-talk between commensal bacteria that inducing immune tolerance and host. Therefore, these studies provide a potential approach to improve immune-mediated diseases such as allergy, based on gut microbial alteration.

## Alleviation of Probiotics on AD Clinical Manifestation

As mentioned earlier, the onset and development of AD are closely associated with gut microbial alterations, and beneficial bacteria such as *Bifidobacterium* and *Lactobacillus* are in shortage in patients. Probiotics consumption may be an effective alternative to supply beneficial bacteria and restore intestinal dysfunction. The gut microbial environment can be reshaped with long-term consumption of probiotics and contributes to the balance of gut microbiota and systemic immune responses. Probiotics promote the synthesis of nutrients such as amino acids and vitamins in the host and increase the content of SCFA in the intestinal lumen. Especially, SCFA including acetate, propionate, and butyrate leads to an intestinal environment with a low pH value to inhibit the growth of pathogens. Additionally, probiotics compete against pathogens including competition for the nutrient substrates and ecological niches, and these interactions contribute to suppression for the excess proliferation of pathogens in the intestine. Therefore, probiotics may alleviate AD clinical manifestation *via* affecting the gut microbial composition, metabolic functions, and immune responses. **Table 2** shows the effects of probiotics on clinical manifestations of patients from pregnant, infant, children to adult and the potential to alleviate AD, although there are some controversial outcomes. Most probiotics reduced the SCORAD (scoring atopic dermatitis index) scores and even decreased the risk of developing AD. The controversial conclusions are associated with many factors such as environment and diet, and in the future, larger samples and more precise experimental design are necessary for clinical trials to verify the effectiveness of probiotics on AD.

**Table 2:** Effects of probiotics on the clinical manifestations of AD in the different crowd.

Probiotics	Participants	Outcome	Reference
*B. breve* M-16V and *B. longum* BB536	Pregnant women; N=130	Probiotics significantly reduced the risk of developing eczema and AD	(95)
*L. rhamnosus* GG, *B. animalis* subsp. *lactis* Bb-12, and *L. acidophilus* La-5	Pregnant women; N=415	Probiotic consumption significantly decreased the proportion of Th22 cells and prevented AD in their offspring	(96)
*Lactobacillus* GG ATCC53103	Pregnant women with a family history of allergy; N=105	*Lactobacillus* GG neither reduced the incidence of AD nor altered the severity of AD	(97)
*L. rhamnosus* GG, *L. acidophilus* La-5, and *B. animalis* subsp. *lactis* Bb-12	Pregnant women; N=415	Probiotics reduced the cumulative incidence of AD but did not affect atopic sensitization	(98)
*Bifidobacterium infantis*, *Streptococcus thermophilus*, and *Bifidobacterium lactis*	Preterm infants; N=1099	Probiotics did not affect the incidence of allergic diseases and atopic sensitization	(99)
*L. rhamnosus* HN001	Infants N=474	*L. rhamnosus* HN001 exerted the protective effect against eczema when given for the first 2 years only, extend to at least 4 years of age	(100)
*B. breve* M-16V and oligosaccharide mixture	Infants aged <7 months with atopic dermatitis; N=90	No effect on AD markers	(101)
*L. rhamnosus* MP108	Children aged 4-48 months with AD; N=66	*L. rhamnosus* MP108 decreased the SCORAD socres	(102)
*L. acidophilus* DDS-1, *B. lactis* UABLA-12 with fructooligosaccharide	Children aged 1-3 years with moderate-to-severe AD; N=90	The clinical improvement was associated with the administration of the probiotic mixture	(103)
*L. plantarum* CJLP133	Children aged 12 months to 13 years; N=118	*L. plantarum* CJLP133 decreased the SCORAD score and total eosinophil count. IFN-γ and IL-4 were significantly reduced compared to baseline measurements	(104)
*L. paracasei* and *L. fermentum*	children aged 1-18 years with moderate-to-severe AD	Probiotics significantly improved the clinical symptoms of AD	(105)
*Lactobacillus pentosus*	Children aged 2-13 years; N=82	Probiotic significantly reduced the SCORAD scores, but the improvement of clinical symptoms had no difference in probiotic and placebo groups	(106)
*Bifidobacterium lactis* CECT 8145, *B. longum* CECT 7347, and *Lactobacillus casei* CECT 9104	Children aged 4 to 17 years with moderate AD; N=50	The SCORAD index and the use of topical steroids were significantly reduced in the probiotic group compared with the control group	(107)
*B. animalis* subsp *lactis* LKM512	Adult patients N=44	*B. animalis* subsp *lactis* LKM512 decreased itch and dermatology specific quality of life scores *via* kynurenic acid of tryptophan metabolism	(108)
Heat-killed *L. paracasei* K71	Adult patients N=34	*L. paracasei* K71 significantly reduced the skin severity scores	(109)

### Regulation of Probiotics on Immune Responses in AD

Based on the “hygiene hypothesis”, bacterial stimulation is required for the maturation of the gut immune system in early life. Most probiotics, derived from the commensal bacteria in the intestine, have been demonstrated to contribute to education for immune tolerance and maintenance of the intestinal immune responses. Immunoglobulin (Ig) A is a crucial antibacterial protein in the intestinal mucosal defense. It blocks pathogens to adhere the intestinal epithelium and increases bacterial entrapment in mucus (110). *Bifidobacterium* is known to stimulate Peyer’s patches to induce IgA production and maintain the integrity of the gut barrier. Administration of *Lactobacillus GG* and *Saccharomyces boulardii* affects the cytokine release and mucosal milieu, and this increases IgA production in the intestine (111). Regulation of the balance between Th1- and Th2-type immune responses is one way to improve clinical symptoms in allergic diseases. *B. animalis* subspecies *lactis* Bb12 increased IgA response in serum and IgG1 and IgG2 response in the ileal fluid in *Ascaris suum* infected pigs (112). *B. animalis* subspecies *lactis* Bb12 treatment improved expression of genes related to Th1/Th2 cells, inflammatory cells, Treg, and physiological function in the gut and reduced Th2 type immune responses. In β-lactoglobulin-induced allergic mice, *L. plantarum* ZDY2013, *L. plantarum* WLPL04 and *L. rhamnosus* GG increased Th1 cells differentiation and inhibited the Th2-biased immune response (113). Furthermore, Treg differentiation not only regulates Th1/Th2 immune balance but suppresses Th17-biased response. *L. paracasei* KBL382 significantly improved the pathological features and altered the gut microbial composition in AD mice (114). It regulated immune balance *via* increasing the expression of IL-10 and transforming growth factor-β and enhancing the differentiation of CD4+ CD25+ Foxp3+ Treg in mesenteric lymph nodes. *L. sakei* WIKIM30 enhanced Treg differentiation in mesenteric lymph nodes *via* inducing DCs tolerance and ameliorated AD-like skin lesions (11). It increased the proportion of *Ruminococcus*, which was positively associated with Treg-related immune responses and might contribute to the alleviation of AD.

Probiotics also contribute to the reduction in the expression of pro-inflammatory cytokines such as IL-13, thymic stromal lymphopoietin (TSLP), and IL-5. The differentiation of eosinophils is closely associated with allergic diseases such as AD, but IL-5 is the critical cytokine to increase the development and survival of eosinophils (115). *L. chungangensis* CAU 28(T) significantly reduced the expression of IL-5, TNF-α, and thymus- and activation-regulated chemokine and alleviated the inflammatory infiltration in AD mice (116). IL-13, like the IL-4, is the key driver to activate Th2-type immune response and shares a common receptor subunit with IL-4. IL-13 and IL-4 bind receptors to activate JAK -STAT6 (Janas kinase-signal transducers and activators of transcription 6) pathways and lead to the decrease in the expression of structural protein such as FLG, involucrin, and lipid composition in the skin (117). Tralokinumab, a monoclonal antibody to neutralize IL-13, had been reported to improve the clinical in adults with AD in randomized, double-blind, multicenter, and placebo-controlled phase III trials (118). *Pediococcus acidilactici* intake reduced the mRNA expression of IL-4, TNF-α, and IL-13 in dorsal skin and improved the clinical severity of AD (119). Levels of TSLP are high in the lesions of AD patients and TSLP is a key protein in the development of AD (120). TSLP is expressed by epithelial cells of the gut, lung, and skin and increases Th2 cell differentiation and Th2-type inflammation through interacting with immune cells such as DCs, natural killer T cells, and CD4+ T cells (121). In a murine model with AD, skin-specific overexpression of TSLP led to the increases in Th2 CD4+ T cells and serum IgE levels (122). Tezepelumab is a monoclonal antibody targeting TSLP and has been reported to treat AD. In a phase 2a study, tezepelumab plus topical corticosteroids (TCS) treatment resulted in a 64.7% reduction in the eczema area and severity index *versus* 48.2% of that in the placebo plus TCS treatment (123). *L. rhamnosus* Lcr35 significantly reduced the expression of IL-4 and TSLP and prevented the development of AD (124). Collectively, probiotics have the great potential to modulate the immune function in AD and may be a microbial alternative strategy to improve AD.

### The Potential Effective Substances of Probiotics to Attenuate AD

**Figure 2:**
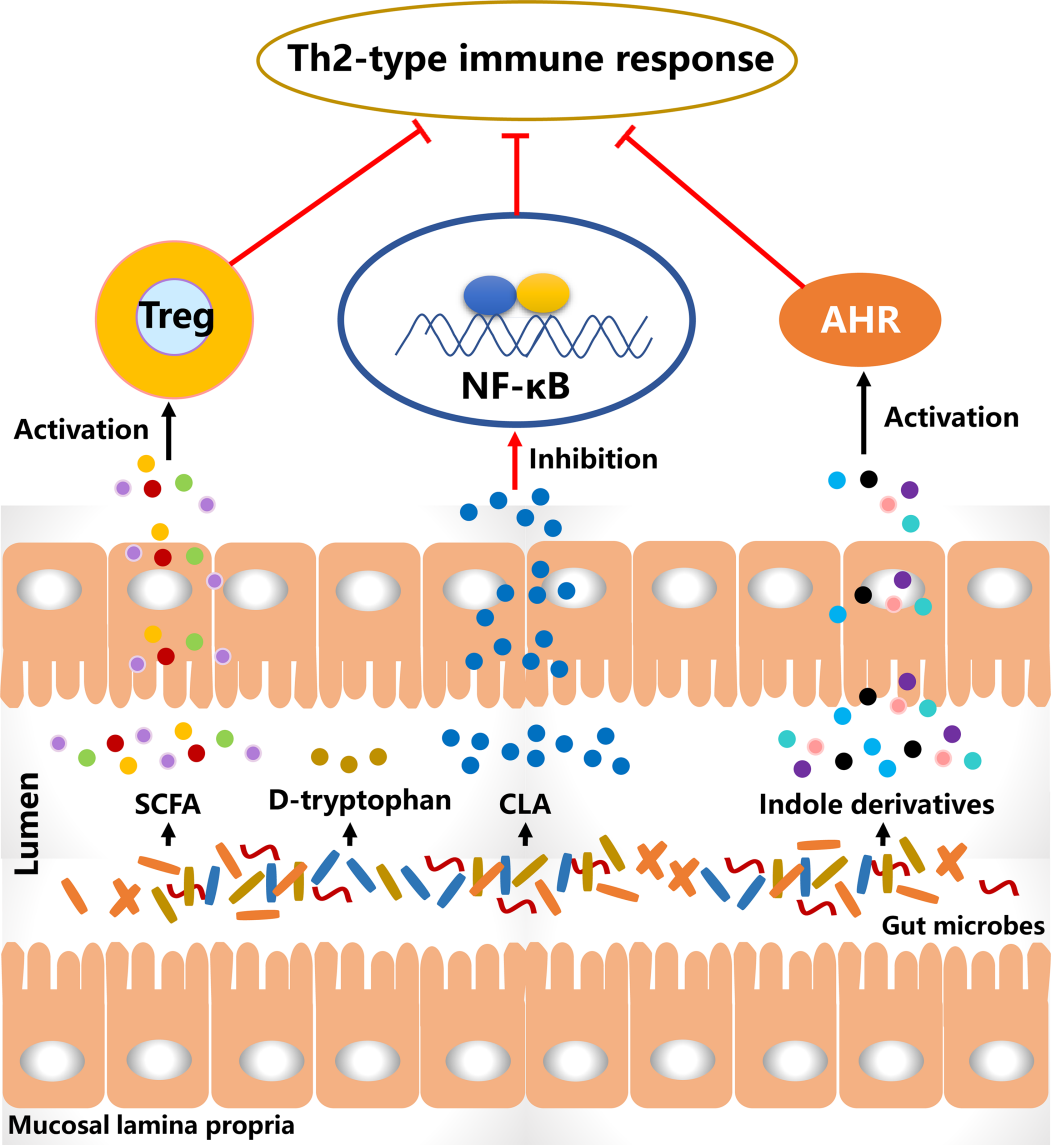
The diagram of **t**he potential effective substances for suppressing Th2-type immune responses. CLA, conjugated linoleic acid; SCFA, short-chain fatty acid; Treg, regulatory T cells.

Probiotics alter the gut microbial composition and simultaneously affect their metabolic activities that may lead to a decreased risk for allergy (**Figure 2**). The metabolites of *B. breve* C50 and *Streptococcus thermophilus* 065 increased the proportion of CD4+ and CD8+ T cells secreting Th1-type cytokine IFN-γ and restored Th1/Th2 immune balance in IL-10-deficient mice (125). Bacteriocins of *B. animalis* subspecies *lactis* Bb12 and *B. longum* Bb46 significantly inhibited the growth of *S. aureus* and *E. coli* in the intestine (126), and these harmful bacteria were associated with the development of AD and the proportion of them was increased in patients. SCFA is produced by the gut microbial fermentation of indigestible carbohydrates and is closely associated with the alleviation of AD clinical manifestations. Furthermore, SCFA has been demonstrated to regulate the size and function of the Treg pool in the intestine (127). In a cohort study, the severity of AD was negative with the proportion of butyrate-producing bacteria in infants and suggesting that butyrate had a potential role in improving AD symptoms (128). High levels of propionate and butyrate in feces reduced atopic sensitization in early life and administration of butyrate decreased the severity of allergic inflammation in mice (129). Antibiotics-induced gut microbial dysbiosis resulted in a decrease in SCFA production and an increase in the levels of inflammatory cells, and these alterations were highly associated with aggravated AD-like skin lesions (130). However, fecal microbial transplantation significantly reduced the clinical score of AD-like lesions *via* increasing SCFA levels and regulating the numbers of immune cells. SCFA contributes to the balance of gut microbiota and is closely associated with levels of immune cells. Therefore, increasing SCFA production in the intestine *via* probiotics consumption may be an effective way to alleviate AD-like symptoms.

D-tryptophan, as a metabolite of *Bifidobacterium*, *Lactobacillus* and *Lactococcus*, suppressed the expression of Th2-associated CCL17 in KM-H2 cells (131). It significantly increased IL-10 production and decreased IL-12, IL-5 and IFN-g in human DCs. After supplementation with D-tryptophan in mice with allergic airway inflammation, the clinical manifestations were alleviated and Th2-biased immune responses were significantly reversed. Conjugated linoleic acid (CLA), as a natural unsaturated fatty acid, can inhibit the release of histamine, which induces an increase in vascular permeability and is associated with the development of AD. *Bifidobacterium*, *Lactobacillus* and *Roseburia* spp. metabolize polyunsaturated fatty acids including omega-3 and omega-6 fatty acids to CLA (132). *B. breve* and *B. pseudocatenulatum* are CLA-producing bacteria and have been reported to alleviate colitis *via* modulating gut microbiota and TLR4/NF-κB signaling (133, 134). It is suggested that probiotics consumption increases CLA production in the intestine and affects the systemic immune responses. *L. plantarum* JBCC105645 and JBCC105683, isolated from the salted fermented seafood according to CLA-producing activity, significantly alleviated the pathological symptoms of AD *via* reducing IL-4 levels and increasing IFN-γ levels (135). This suggested that CLA might be the material basis of probiotics to alleviate AD. Oral administration of CLA significantly attenuated AD-like skin lesions *via* inhibition of COX-2/5-LOX and TLR4/NF-κB signaling pathways (136). The results showed with the anti-inflammatory effect of CLA had a strong potential to alleviate AD.

Aryl hydrocarbon receptor (AHR) has been reported to be closely associated with the development of AD (137). Indole-3-aldehyde (IAld), a metabolite of tryptophan, was lower in AD lesional skin than that of healthy controls and significantly alleviated skin inflammation *via* activating AHR (138). Coal tar is usually used to improve the clinical symptoms of AD. In the skin models with primary keratinocytes, it activated AHR to induce epidermal differentiation and interfered with Th2 cytokine signaling (139). These results suggest the activation of AHR plays an important role in the treatment of AD. Indole derivatives including IAld, tryptamine, indole acetic acid, indole-3-acetaldehyde, indole acrylic acid, and indole-3-propionic acid, are metabolites from tryptophan metabolism of gut microbiota and have been demonstrated as the ligands to activate AHR (140). In a study involving the gut-brain axis, gut microbial metabolites of tryptophan affected the activation of microglia and modulated the central nervous system inflammation *via* a mechanism mediated by AHR (141). Additionally, the metabolites of tryptophan such as indoxyl sulfate and indole-3-propionic acid have been found in blood circulation (142). This suggests that gut microbiota-produced ligands of AHR have the potential to regulate systemic inflammation, including skin inflammation. In a meta-analysis, probiotics consumption significantly regulated the ratio of kynurenine: tryptophan and mediated the tryptophan metabolism (143). Therefore, these studies imply that probiotics regulate tryptophan metabolism in the intestine, and the metabolites as AHR ligands may mediate skin inflammation *via* AHR signaling.

There are some limitations about the effects of probiotics on the alleviation of AD in this review. The effectiveness of probiotics on the improvement of clinical symptoms of AD needs the larger scale and more rigorous clinical trials to demonstrate in different groups of patients stratified by age, sex, and concurrent diseases. The interactions between probiotics and gut microbiota are complex and lead to difficulties in revealing the precise alleviating mechanisms on AD. Furthermore, the immunomodulation of probiotics is strain-specific and they may activate different signaling pathways to improve the clinical manifestations of AD. The substance basis of probiotics to alleviate AD is still to be elucidated whether it is from the component of probiotic itself and the metabolites from probiotic or gut microbiota.

## Concluding Remarks

In summary, although the cross-talk mechanism between gut microbiota and skin needs to be explored, the gut microbiota is closely associated with dermatology and may serve as a target for the prevention and treatment of AD. Probiotics supplementation alters the intestinal environment, including modulating gut microbial composition, preventing pathogens colonization, affecting bacterial metabolism, and restoring immune balance. These alterations may contribute to the decrease in inflammation and improvement of clinical manifestation in AD. Although the effects of probiotics on AD have been investigated in numerous clinical trials, the effective substance basis of probiotics to alleviate AD remains unclear. To precisely manipulate gut microbiota to attenuate clinical manifestation of AD, the mechanism of interactions between probiotics, gut microbiota, and skin needs to be elucidated. Combination with metatranscriptomics, metagenomics and metabolomics, effects of probiotics on functional gene alteration, specific gut microbe, metabolic pathway, and specific metabolite could be revealed in the future and overall analyze the alleviating mechanism of probiotics targeting the gut microbiota. Future results of probiotic clinical trials on AD may support a microbiome replacement strategy.

## Author Contributions

ZF: writing-original draft. LL: editing. HZ, JZ, and WC: writing-review and funding acquisition. WL: wring-review and editing, project administration, and funding acquisition. All authors contributed to the article and approved the submitted version.

## Funding

This research was supported by the National Natural Science Foundation of China (No. 31820103010), the national first-class discipline program of Food Science and Technology (No. JUFSTR20180102), the Fundamental Research Funds for the Central Universities (No. JUSRP51903B), the Postdoctoral Research Funding Scheme of Jiangsu Province (No. 2021K018A).

## Conflict of Interest

The authors declare that the research was conducted in the absence of any commercial or financial relationships that could be construed as a potential conflict of interest.
